# Estimation of population affinity using proximal femoral measurements based on computed tomographic images in the Japanese and western Australian populations

**DOI:** 10.1007/s00414-024-03257-5

**Published:** 2024-05-20

**Authors:** Suguru Torimitsu, Akari Nakazawa, Ambika Flavel, Lauren Swift, Yohsuke Makino, Hirotaro Iwase, Daniel Franklin

**Affiliations:** 1 Centre for Forensic Anthropology, University of Western Australia, Crawley, WA 6009 Australia; 2 Department of Forensic Medicine, Graduate School of Medicine, The University of Tokyo, Tokyo, 113-0033 Japan; 3 Department of Obstetrics and Gynecology, Graduate School of Medicine, The University of Tokyo, Tokyo, 113- 8655 Japan

**Keywords:** Ancestry estimation, Femora, Computed tomography, Japanese, Western Australian

## Abstract

The present study analyzes morphological differences femora of contemporary Japanese and Western Australian individuals and investigates the feasibility of population affinity estimation based on computed tomographic (CT) data. The latter is deemed to be of practical importance because most anthropological methods rely on the assessment of aspects of skull morphology, which when damaged and/or unavailable, often hampers attempts to estimate population affinity. The study sample comprised CT scans of 297 (146 females; 151 males) Japanese and 330 (145 females; 185 males) Western Australian adult individuals. A total of 10 measurements were acquired in two-dimensional CT images of the left and right femora; two machine learning methods (random forest modeling [RFM]) and support vector machine [SVM]) were then applied for population affinity classification. The accuracy of the two-way (sex-specific and sex-mixed) model was between 71.38 and 82.07% and 76.09–86.09% for RFM and SVM, respectively. Sex-specific (female and male) models were slightly more accurate compared to the sex-mixed models; there were no considerable differences in the correct classification rates between the female- and male-specific models. All the classification accuracies were higher in the Western Australian population, except for the male model using SVM. The four-way sex and population affinity model had an overall classification accuracy of 74.96% and 79.11% for RFM and SVM, respectively. The Western Australian females had the lowest correct classification rate followed by the Japanese males. Our data indicate that femoral measurements may be particularly useful for classification of Japanese and Western Australian individuals.

## Introduction

The identification of human skeletal remains, especially those that are significantly damaged and/or poorly preserved, is important in forensic anthropological practice [1]. The availability of multiple reliable methods to facilitate an anthropological assessment (e.g., biological profile) increases the likelihood of being able to identify unknown individuals from such remains [2]. Population affinity is a statistical approach based on underlying population structure that allows us to understand how microevolutionary forces work in concert with historical events to shape the diversity of modern humans [3]. Although it is an accepted fact that population affinity estimation, in conjunction with other parameters such as age-at death, is essential to the human identification process, it is one of the most difficult (and controversial) parameters of the biological profile [4, 5].

Because of known variations in cranial morphology between different geographic populations, numerous approaches to the estimation of population affinity based on the assessment of cranial feature, both morphoscopic and morphometric, are commonly applied by forensic anthropologists [6, 7]. In cases where the skull is unavailable or non-diagnostic due to poor preservation, the postcranial skeleton affords the only opportunity to morphologically evaluate population affinity. However, the methods for estimating population affinity based on the postcranial skeleton are less commonly applied because of relatively low ancestral variation that flows into poor predictive accuracy. The femur, especially the proximal region, is one of only a few postcranial bones that has previously been shown to present significant morphological variances relative to population affinity [8–13].

At present, there is a paucity of published research concerning estimating population affinity based on the analysis of the fragmentary femur [2, 14, 15]. Further, even though there are 8,443 Japanese residents in Western Australia in 2023 [16], no research has hitherto compared the morphology of Japanese and Western Australian femora. Asian, including Japanese, populations include individuals of different ethnic groups and from countries exhibiting evident phenotypic diversity in body size and shape [17]. Australia is a multicultural country with dynamic demographics, including many migrants from Southeast Asia [18]. In addition, there are approximately 750 unidentified human remains and 2,500 long-term missing persons cases in Australia in 2022 [19]. Therefore, an understanding of how such groups vary morphologically affords opportunity to further understand the utility of estimating ancestry in bones other than the skull, which has practical benefit in a forensic context.

Computed tomography (CT) provides a time-saving alternative to physical forensic examination because it can visualize a high level of detail in bone structures without requiring the removal of soft tissue, thus also protecting the remains from further invasive manipulation [17, 20]. In addition, two- and three-dimensional (2D; 3D) reconstruction of CT images can be analyzed with an appropriate level of reproducibility and accuracy; medical imaging thus provides an adequate proxy for physical remains to develop forensic standards that facilitate biological profiling of unknown skeletal remains [21].

As there is little extant research quantifying population affinity based on femoral measurements acquired in CT images [15, 22], the aims of the present study are as follows: (i) quantify morphological differences in the femora of contemporary Japanese and Western Australian individuals; and (ii) assess the accuracy of estimating population affinity based on the latter bone.

## Materials and methods

### Materials

#### Japanese population

The sample comprised postmortem CT (PMCT) scans of 297 adult corpses (146 female, mean age = 50.32 ± 16.75 years; 151 male, mean age = 48.83 ± 15.23 years) obtained from the Department of Forensic Medicine at the University of Tokyo between August 2017 and June 2022. The estimated postmortem interval for all the subjects was < 14 days. Exclusion criteria were femoral fractures, burn injuries, bone implant, visible degradation, pathologies, or anomalies potentially affecting normal measurements. The study protocol was approved by the Ethics Committee of the University of Tokyo (2121264NI).

#### Western Australian population

The sample comprised MDCT scans of 330 individuals age 18 years and older (145 female, mean age = 46.18 ± 12.60 years; 185 male, mean age = 44.35 ± 13.52 years) who presented at one of the major Western Australian hospitals (Perth region) for clinical evaluation between May 2007 and June 2012. In accordance with the National Statement on Ethical Conduct in Human Research (National Statement), the scans were anonymized, retaining only sex and age information. Although specific information on the ethnicity of each individual was not maintained in the patient data, the entire sample was taken as representative of a “typical” Western Australian population [23]. The largest proportion of the contemporary Australian population comprises individuals who identify as being of English (33.0%), Australian (29.9%), Irish (9.5%), Scottish (8.6%) and Chinese (5.5%) ethnicity; people who identify as Aboriginal and/or Torres Strait Islander decent comprise 3.2% of the total Australian population [24]. The exclusion criteria were the same as written for the Japanese population. Research ethics approval was granted by the Human Research Ethics Committee of the University of Western Australia (2020/ET000038).

### Methods

For the Japanese subjects, PMCT scanning was performed with a 16-row detector CT system (Eclos; Fujifilm Healthcare Corporation, Tokyo, Japan). The scanning protocol was as follows: collimation of 1.25 mm, reconstruction interval of 1.25 mm, tube voltage of 120 kV, and tube current of 200 mA. For Western Australian subjects, MDCT imaging was performed using a 64-slice CT scanner (Brilliance; Phillips Healthcare, NSW, Australia) with an average slice thickness of 1.03 mm, tube voltage of 100–140 kV, and automatic tube current modulation. The reconstructions were performed per the original CT scan slice thickness.

**Fig. 1:**
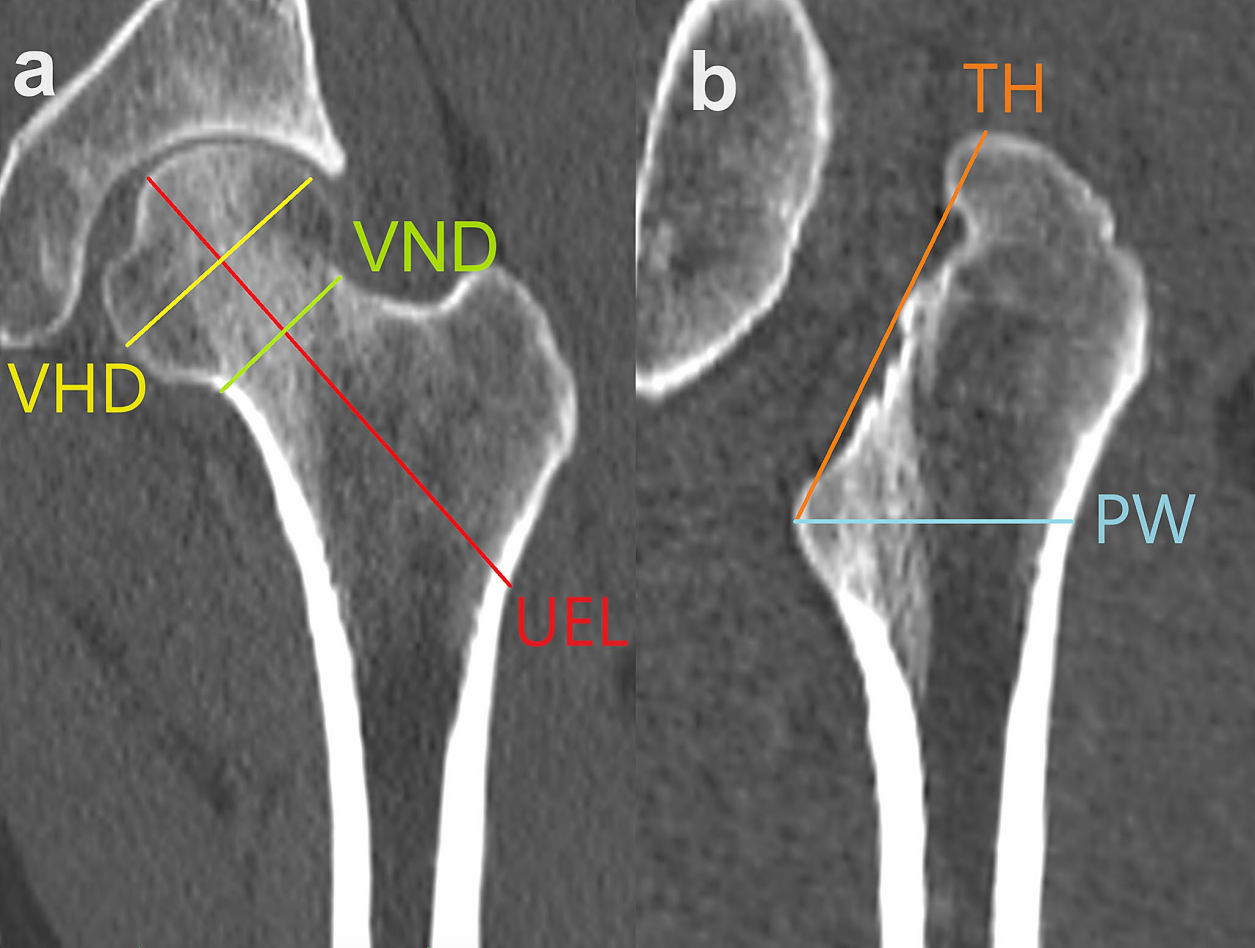
Two-dimensional computed tomography images showing five femoral measurements (see Table 1 for definition): (a) Upper epiphyseal length (UEL), vertical head diameter (VHD), and vertical neck diameter (VND) on a coronal oblique plane; (b) trochanter height (TH) and proximal width (PW) on a plane passing through PW and the superior point of the greater trochanter

Image data processing was performed on a workstation (OsiriX MD version 11.0.2; Pixmeo SARL, Geneva, Switzerland). Bone kernel was acquired and sent to the workstation. 2D CT images were viewed using a window width and level of 1500 and 300 HU, respectively. Five measurements (Table 1) were performed on left and right proximal femur, respectively. For the measurements of the upper epiphyseal length (UEL), vertical head diameter (VHD), and vertical neck diameter (VND), the coronal oblique plane reconstructed along the middle of the femoral head, neck, and trochanter was used [25] (Fig. 1a). The proximal width (PW) was confirmed in a horizontal section, and the trochanter height (TH) and PW were measured on a plane passing through PW and the superior point of the greater trochanter (Fig. 1b). The measurements were performed manually on CT images to the nearest 0.1 mm.

**Table 1:** Definitions of the femoral measurements

Measurement	Definition
Upper epiphyseal length (UEL)	Maximum upper epiphyseal length measured along the elongated neck axis from the most medial point on the femoral head to the lateral surface of the femur [27]
Vertical head diameter (VHD)	Maximum rectilinear vertical femoral head diameter measured perpendicular to the neck axis [27]
Vertical neck diameter (VND)	Minimum rectilinear cranio-caudal diameter of the neck [27]
Trochanter height (TH)	Distance between the superior point of the greater trochanter and the most medially projecting point on the lesser trochanter
Proximal width (PW)	Maximum diameter of the horizontal section including the most medially projecting point on the lesser trochanter

Inter- (ST) and intra-observer reliability (AN) was assessed with repeated measurements taken from six subjects that were randomly selected from the sample. All ten femoral measurements were performed on each of the six subjects, and this process was repeated six times, with a minimum of two-day intervals. Subsequently, the relative technical error of measurement (rTEM) and coefficient of reliability (*R*) were calculated. The acceptable rTEM range was taken to be < 5% [26–28] and *R* values > 0.75 were considered sufficiently precise [29, 30].

Descriptive statistics including ranges, mean, standard deviation and median for each set of measurements for both sexes and populations were calculated. The Brunner–Munzel test was used to determine if significant differences existed between the two groups; a *p* value of < 0.05 was considered statistically significant. Because all measurements were localized and related to the proximal femur, multicollinearity was verified by calculating the variance inflation factor (VIF). The analyses were performed using Excel (Microsoft Office 2019, Microsoft, Redmond, Washington, USA).

Two machine learning methods were used for population affinity classification: **(i)** random forest modeling (RFM) which is the process of repeatedly testing randomly drawn samples from the original training data (bootstraps), iterating the process to refine the model with multiple trees and aggregating the models trained on each bootstrap (bagging) [15, 31], and **(ii)** support vector machine (SVM) which generates classification rules by maximizing the margin between the two groups using data located at the edge of the multivariate space (the intersection of two groups) [32, 33].

Separate analyses using machine learning models were performed to classify the remains according to **(i)** two-way models distinguished by sex-specific (female and male) and sex-mixed population affinity; and **(ii)** a four-way model distinguished simultaneously by population affinity and sex. For the RFM, the random forest feature importance during the analysis was also calculated. Machine learning performance was analyzed using R 4.3.2 (R Foundation for Statistical Computing, Vienna, Austria) with the “randomForest” and “e1071” packages [34, 35].

## Results

The intra-observer rTEM and *R* values ranged between 0.58 and 1.84%, and 0.870–0.994, respectively. Inter-observer agreement presented with similar variation, with the rTEM and *R* values ranging between 0.58 and 1.87%, and 0.860–0.994, respectively (Table 2).

**Table 2:** Relative technical error of measurements (rTEM, %) and coefficient of reliability (*R*)

Measurement	Intra-observer error			Inter-observer error	
	rTEM	*R*		rTEM	*R*
LUEL	0.62	0.989		0.58	0.990
LVHD	1.13	0.870		1.15	0.860
LVND	1.84	0.924		1.87	0.919
LTH	0.87	0.959		0.85	0.961
LPW	1.09	0.983		1.13	0.981
RUEL	0.58	0.994		0.60	0.994
RVHD	1.29	0.900		1.20	0.916
RVND	1.78	0.925		1.80	0.917
RTH	0.81	0.934		0.84	0.930
RPW	1.22	0.963		1.15	0.966

No significant age differences were found between the populations in both sexes (male, *p* = 0.081; female, *p* = 0.059).

The range, mean, standard deviation, and median values of the 10 measurements (five measurements on each side) for both sexes are shown in Tables 3 and 4. Regarding the female sample, all of the measurements were significantly larger in the Western Australian compared to the Japanese population. In considering the male sample, all measurements except for LTH and RTH were statistically significantly larger in the Western Australian population; no significant population affinity differences were observed LTH and RTH. The VIF values for each of the 10 measurements ranged from 1.87 to 6.67.

**Table 3:** Descriptive statistics of 10 femoral measurements (five measurements on each side) for the female sample

Measurement	Japanese (*n* = 146)				Western Australian (*n* = 145)		
	Range	Mean ± SD	Median		Range	Mean ± SD	Median
LUEL (mm)	7.39–9.79	8.63 ± 0.46	8.66		8.30–10.42	9.32 ± 0.42	9.27*
LVHD (mm)	3.41–4.79	4.20 ± 0.21	4.22		3.98–4.79	4.37 ± 0.18	4.37*
LVND (mm)	2.14–3.23	2.72 ± 0.22	2.74		2.61–3.36	2.93 ± 0.18	2.94*
LTH (mm)	4.30–7.55	6.34 ± 0.47	6.31		5.59–7.59	6.60 ± 0.43	6.58*
LPW (mm)	3.54–4.89	4.26 ± 0.28	4.25		3.72–5.29	4.47 ± 0.32	4.46*
RUEL (mm)	7.39–9.40	8.63 ± 0.44	8.70		8.54–10.13	9.18 ± 0.38	9.16*
RVHD (mm)	3.62–4.65	4.22 ± 0.20	4.22		4.05–4.89	4.39 ± 0.18	4.39*
RVND (mm)	2.14–3.25	2.72 ± 0.21	2.75		2.55–3.33	2.93 ± 0.19	2.93*
RTH (mm)	4.18–7.64	6.34 ± 0.50	6.32		5.47–7.70	6.57 ± 0.47	6.59*
RPW (mm)	3.59–5.03	4.29 ± 0.27	4.29		3.86–5.34	4.50 ± 0.32	4.48*

SD, Standard deviation; * Significantly larger than that of Japanese (*p* < 0.05 using Brunner–Munzel test)

**Table 4:** Descriptive statistics of 10 femoral measurements (five measurements on each side) for the male sample

Measurement	Japanese (*n* = 151)				Western Australian (*n* = 185)		
	Range	Mean ± SD^a^	Median		Range	Mean ± SD	Median
LUEL (mm)	9.30–10.76	9.82 ± 0.36	9.76		9.36–11.73	10.42 ± 0.56	10.43*
LVHD (mm)	4.41–5.46	4.83 ± 0.19	4.82		4.36–5.71	4.94 ± 0.25	4.91*
LVND (mm)	2.81–3.89	3.22 ± 0.19	3.18		2.89–4.05	3.39 ± 0.23	3.38*
LTH (mm)	6.23–8.16	7.24 ± 0.42	7.25		6.26–9.04	7.34 ± 0.50	7.31
LPW (mm)	4.30–5.47	4.84 ± 0.27	4.82		4.33–6.28	4.99 ± 0.36	4.93*
RUEL (mm)	9.30–11.00	9.85 ± 0.37	9.78		9.39–12.14	10.38 ± 0.54	10.32*
RVHD (mm)	4.42–5.41	4.85 ± 0.19	4.82		4.30–5.83	4.91 ± 0.26	4.88*
RVND (mm)	2.86–3.83	3.22 ± 0.20	3.21		2.88–3.98	3.37 ± 0.23	3.36*
RTH (mm)	6.27–8.45	7.28 ± 0.43	7.27		6.21–8.86	7.33 ± 0.49	7.31
RPW (mm)	4.34–5.47	4.87 ± 0.26	4.87		4.30–6.22	5.01 ± 0.37	4.98*

SD, Standard deviation; * Significantly larger than that of Japanese (*p* < 0.05 using Brunner–Munzel test)

The results of the machine learning models are presented in Tables 5 and 6. The accuracy of the two-way models ranged from 71.38 to 82.07% and 76.09–86.09% for RFM and SVM, respectively. The sex-specific models had slightly higher correct classification rates than the sex-mixed models. No considerable differences in the correct population affinity classification rates were observed between the female- and male-specific models. All the correct classification rates were higher in the Western Australian sample except for the male model using SVM.

**Table 5:** Classification matrix showing the classification of groups according to population affinity (two-way models)

Sex	Group	RFM				SVM		
		JP	WA	% Correct		JP	WA	% Correct
Female	JP	112	34	76.71%		120	26	82.19%
	WA	26	119	82.07%		23	122	84.13%
	All			79.38%				83.16%
Male	JP	118	33	78.15%		130	21	86.09%
	WA	37	148	80.00%		29	156	84.32%
	All			79.17%				85.12%
Sex-mixed	JP	212	85	71.38%		226	71	76.09%
	WA	68	262	79.39%		54	276	83.64%
	All			75.60%				80.06%

RFM, random forest modeling; SVM, support vector machine; JP, Japanese; WA, Western Australian

**Table 6:** Classification matrix showing the classification of groups according to population affinity and sex (four-way models)

Group	RFM						SVM				
	JPF	JPM	WAF	WAM	% Correct		JPF	JPM	WAF	WAM	% Correct
JPF	113	0	33	0	77.40%		121	0	25	0	82.88%
JPM	0	111	8	32	73.51%		0	120	7	24	79.47%
WAF	27	10	102	6	70.34%		24	9	105	7	72.41%
WAM	0	37	4	144	77.84%		0	31	4	150	81.08%
All					74.96%						79.11%

JPF, Japanese female; JPM, Japanese male; WAF, Western Australian female, WAM, Western Australian male

The four-way model revealed overall classification accuracy values of 74.96% and 79.11% for RFM and SVM, respectively; these values were slightly smaller than those in the two-way models. Correct classification according to population was lowest in Western Australian female sample, followed by the Japanese male sample.

**Fig. 2:**
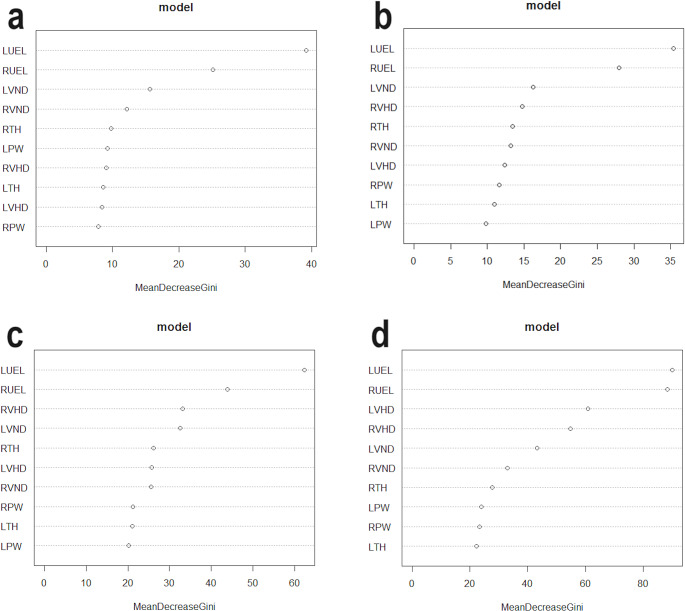
Random forest feature importance (mean decrease Gini) for the response variable: (a) the two-way female model, (b) the two-way male model, (c) the two-way sex-mixed model, and (d) the four-way population affinity and sex model

The random forest feature importance showed that left and right UELs were the strongest weighted measurements for correct classifications (express the greatest population variance) (Table 7; Fig. 2).

**Table 7:** Random forest feature importance (mean decrease Gini) in each model for the response variable

Variable	Mean decrease Gini						
	2-way female model		2-way male model		2-way sex-mixed model		4-way population affinity and sex model
LUEL	39.20		35.39		62.38		90.11
LVHD	8.44		12.35		25.70		60.87
LVND	15.61		16.25		32.52		43.30
LTH	8.58		10.99		21.11		22.35
LPW	9.23		9.85		20.24		23.97
RUEL	25.11		27.96		43.96		88.51
RVHD	9.03		14.79		33.22		54.73
RVND	12.19		13.19		25.52		32.88
RTH	9.75		13.46		26.19		27.76
RPW	7.90		11.61		21.22		23.36

## Discussion

In the present study, the intra- and inter-observer errors were small and can be acceptable; the femoral measurement using CT images was thus considered to be precise and reproducible.

Previous studies suggested that femoral neck width increased with age probably due to endocortical resorption from maintaining resistance to loaded stress [22, 36, 37]. However, in this study, there were no significant age differences between the populations in both sexes.

The results of this study demonstrated that significant differences between the Japanese and Western Australian populations in various measurement values. Previous studies [38, 39] reported significant variances in femoral measurements between South African groups (Black, White, and mixed-ancestry). Several studies demonstrated that Asian groups had shorter femoral neck dimensions and thicker cortical bone than other groups [22, 40, 41]. Attia et al. [15] also reported that The Egyptian had shorter femoral necks on average than individuals in other populations studied. Conversely, Chin et al. [40] suggested that premenopausal Polynesian females had longer femoral necks than European and other ethnic groups. The latter data combined clearly suggest that there may be considerable differences in femoral measurements between different populations.

Colman et al. [25] reported the high VIF values of femoral head measurements in a Dutch population, indicating high levels of multicollinearity. The results of this study also demonstrated some measurements with VIF values more than 6. Thus, there is a need to explore femoral bone measurements with less multicollinearity in the future.

In the present study, the classification accuracy of Japanese and Western Australian individuals to their respective population groups was approximately 80% using RFM and SVM, respectively. Similarly, Attia et al. [15] reported that using RFM and the linear variables based on femoral measurements in Egyptian, Indian, and Greek populations, the overall accuracy reached 83% and 72% for females and males, respectively. Thus, although phenotypic differences between populations are known to be most pronounced in the skull [42], the comprehensive information presented in this study may be particularly useful for forensic investigations where the skull is damaged and unavailable. L’Abbé et al. [43] reported that data on the cranium tend to misclassify individuals according to sex. On the other hand, sexual size dimorphism appears to be more evident in the postcranial skeleton [44], and previous studies have found postcranial elements to be useful in facilitating more accurate sex estimates than the skull [11, 45–47]. Thus, the use of a multifactorial approach provides potential to combine probability and likelihood, to enhance identification efforts, and to achieve greater reliability that is necessary in a forensic context.

The present study demonstrated that performing sex-specific analyses of population affinity improves classification accuracy by reducing group overlap and more effectively separating groups; Attia et al. [15] reported a similar finding. Sex accounts for most of the variation between groups, and population affinity for most of the remaining [48], therefore, when the effect of population-specific sexual dimorphism is removed, the model only has to assess morphological differences associated with population variances.

Previous studies have found that higher classification accuracy was generally achieved with female, compared to male, sex-specific models [15]. Using linear discriminant analysis, Holliday and Falsetti [49] achieved 100% accurate population affinity classification in females, versus 87% of the male training sample for discriminating African-American from European-American skeletons using postcranial measurements. Liebenberg et al. [44] reported higher classification accuracy for South African Black females (70%) relative to males (67%), and for colored females (80%) compared to males (73%). However, in the present study, there was no considerable differences in the correct classification rates between the female- and male-specific models. Similarly, Liebenberg et al. [44] revealed that both females and males were classified equally (93%) among South African whites.

The two-way models applied in the present study demonstrate that correct classification rates were higher for Western Australian individuals except for the male model using SVM. However, regarding the four-way models, the Western Australian female and Japanese male individuals had the lower correct classification rates compared with the Western Australian male and Japanese female samples. These results indicate that the proximal femur of Western Australian female and Japanese male are similar in size, larger than those of Japanese females, and smaller than those of Western Australian males. Therefore, it is suggested that accurate sex determination is quite important for population affinity estimation between Japanese and Western Australians.

In the present study, the UEL was the most accurate variable for discriminating between the two population groups. Christensen et al. [14] also reported that the UEL showed significant differences among all groups (Europeans, Africans, and Asians) and may therefore potentially be used to reliably assess population affinity in unidentified human remains in a forensic context.

In the present study, machine learning methods were used for population affinity classification. Over the last decade, machine learning algorithms have provided new insight into human variation. In addition, they have outperformed traditional classification methods in anthropological research [31, 50]. Recently, researchers have used RFM in both morphoscopic and morphometric approaches to population affinity estimation [31, 32]. RFM can tackle classification and regression tasks in a supervised learning framework, and in terms of unsupervised learning, it can be used for data clustering, missing value imputation, and novelty and outlier detection [30]. The most important advantage of RFM is that it transforms low-bias and high-variance models into low-bias and low-variance models, by training multiple decision trees simultaneously because low variance is the most valuable feature for anthropological applications [51]. In addition, one of the most interesting features is that it does not require cross-validation to obtain unbiased estimates of model performance [30]. Moreover, RFM provides more accurate models than linear discriminant analysis in population affinity estimation (multi-group classification) based on the femoral measurements [15]. However, the classical algorithms have the advantage of being able to create estimation formulae. Therefore, regarding population affinity estimation using the measurements ​​of this study, it is necessary to conduct a comparative study between machine learning algorithms and major classical algorithms in the future. In addition, Attia et al. [15] also reported that classification accuracy varied depending on the type and number of variables used, available skeletal elements, and the specific populations studied. Thus, further investigation of the feasibility of population affinity estimation based on other bones, and/or in other populations, would provide useful information for professional practice.

Previous studies [52–54] demonstrated that the SVM was more effective than RFM in population affinity assessment. Similarly, in the present study, probably due to the relatively small amount of data, SVM showed higher rates of correct classification than RFM. However, no study has used SVM for population affinity estimation based on femoral measurements. Further studies regarding other machine learning methods for population affinity assessment are required to explore this in more detail.

There were several studies on population affinity estimation which have analyzed the femoral data obtained from physical specimens and dual-energy x-ray absorptiometry [2, 14]. Conversely, aside from the work we present here, only one previous study [15] has investigated the feasibility of multiple femoral measurements acquired in CT images to estimate population affinity. CT imaging can reduce the time-consuming and tedious nature of skeletal maceration or the need for physical storage space [55–57]. In addition, CT data is easier to share among institutions in different countries than the physical specimen, which facilitates collection of multi-population data and a deeper understanding of the diversity of femoral morphology.

It is important to acknowledge that the present study had some limitations. First, both PMCT and CT data from surviving patients were used. Although the measurements of human bones are not expected to change dramatically after death, those differences were not assessed in this study. Second, data were collected from two different facilities using 16- and 64-row detector CT systems and under different conditions for reconstructed images. However, it has been empirically demonstrated that variations in the type of CT scanner used, slice thickness, and exposure levels have no significant effect on the acquisition of linear measurement data [56]. Thus, data from a large number of hospitals and departments can be used without concern regarding the accuracy of the virtual models generated. Third, estimating population affinity from the proximal femur alone requires consideration of differences in physical activity, environmental adaptations, genetic origins, and diet [22, 58–61]. However, information on those details was not available in the present study. Finally, estimation formulas are not derived when using machine learning algorithms as in this study; the use of the method in this study at other institutions is limited. In the future, it will be necessary to conduct further comprehensive research and develop software for population affinity estimation which can be applied to forensic investigation.

## Conclusions

This study demonstrated that application of proximal femoral measurements derived in CT images can be used to accurate classify individuals of Japanese and Western Australian origin; this is especially beneficial in a forensic or anthropological context where commonly investigated elements such as the skull are unavailable. Further studies regarding population affinity estimation based on other skeletal measurements and populations should be conducted.
